# Differential Associations of Sports Participation With Self-Rated Health and Depressive Symptoms Among Japanese Adolescents

**DOI:** 10.7759/cureus.43776

**Published:** 2023-08-19

**Authors:** Satoshi Yamaguchi, Yohei Kawasaki, Ayako Oura, Seiji Kimura, Manato Horii, Shotaro Watanabe, Takahisa Sasho, Seiji Ohtori

**Affiliations:** 1 Graduate School of Global and Transdisciplinary Studies, Chiba University, Chiba, JPN; 2 Graduate School of Medical and Pharmaceutical Sciences, Chiba University, Chiba, JPN; 3 Epidemiology and Public Health, Institute for Assistance of Academic and Education, Tokyo, JPN; 4 Center for Preventive Medical Sciences, Chiba University, Chiba, JPN

**Keywords:** sports participation, self-rated health, physical activity, mental health, depression, adolescent

## Abstract

Introduction: We aimed to evaluate the associations of sports participation with self-rated health and depressive symptoms in a nationally representative sample of Japanese adolescents.

Methods: A questionnaire survey was conducted with 1,658 adolescents aged between 12 and 21 years. Sports participation levels were divided into high-frequency, moderate-frequency, low-frequency, and no-participation groups. Self-rated health was evaluated using a four-grade scale. Depressive symptoms were assessed using the Japanese version of the Patient Health Questionnaire-8. Other lifestyle behaviors were also surveyed. The associations of sports participation with self-rated health and depressive symptoms were examined using multiple logistic regression analysis.

Results: The participants in the high- (odds ratio (OR), 0.45) and moderate-frequency (OR, 0.46) groups were less likely to self-report poor health than those in the non-participation group. By contrast, a U-shaped association was found between sports participation and depression, in which the participants in the moderate-frequency group (OR, 0.52) were less likely to have depressive symptoms. The OR for the high- (0.89) and low-frequency (0.91) groups were not significant. Furthermore, eating regular breakfasts, six or more days/week, and having shorter screen times of less than two hours/day were negatively associated with poor self-reported health and depressive symptoms.

Conclusion: Moderate- to high-frequency sports activities are associated with a reduced risk of poor self-rated health among Japanese adolescents. However, only moderate-frequency activities were associated with a reduced risk of depression. Participation in optimal sports activities may effectively lower the risk of poor health in adolescents.

## Introduction

Maintaining adolescent health is gaining attention because adolescents are exposed to health risk factors alongside the changes in social relations and exposure to online interactions [1]. Moreover, health status during adolescence is linked to health in later life [2]. Self-rated health is a widely used outcome to measure the global health status of adolescents [3]. It is a reliable physical and mental health indicator that predicts healthcare utilization, medication, and mortality in early adulthood [2]. Mental health, including depression, is another critical health outcome for adolescents. The prevalence of adolescent mental health problems is 15% in Europe and perhaps even higher in Asian countries [4,5]. Depression is one of the most prevalent mental disorders [6]. Furthermore, the onset of depression is common in adolescents [6]. Therefore, determining the risk and protective factors of these health outcomes provides insights into creating individual intervention strategies and public policy-making to improve adolescent health.

Physical activity is associated with physical and mental health benefits in adolescents [3]. In particular, participation in sports activities is the most popular form of leisure-time physical activity in adolescents [7,8]. Engaging in sports activities also fosters social relationships and self-esteem [9]. Consequently, sports participation has been recommended to improve adolescents’ physical and mental health [9]. In addition to sports participation, various lifestyle behaviors, such as screen time, hours of sleep, and diet, can affect adolescent health [10,11]. These factors are interactive; thus, they should be considered simultaneously [12]. Furthermore, other factors, including age, sex, socioeconomic status, and family status, have been associated with self-rated health and depression in adolescents. Identifying associated multifaceted factors is important because a holistic approach is recommended to improve adolescent health [10].

Previous studies in this field have had several limitations. First, they surveyed either self-rated health or mental health as the outcome, although the impact of sports participation may vary depending on health outcomes of interest [13]. The differential association between sports participation and these outcomes therefore remains unclear. Second, studies on the association between sports participation and adolescent health have been reported in European and North American countries [9]. However, adolescent health, sports participation, and their associations may vary across countries [10]. Therefore, the results of these studies may not be applicable to Japan, where subjective health evaluations are generally lower than those in European countries [14]. Third, several studies have reported self-rated health and physical activity among Japanese adolescents [15-17]; nonetheless, these did not specifically focus on the impact of sports participation. Moreover, the participants of the Japanese studies were recruited from a limited region with narrow demographics [15-17]. Therefore, the results of these studies may not be generalizable to all Japanese adolescents.

In order to address these issues, we aimed to examine the associations among sports participation, self-rated health, and depressive symptoms among Japanese adolescents using a nationally representative sample.

## Materials and methods

Data collection

The Research Ethics Committee of the Graduate School of Medicine, Chiba University, approved this study (approval number: M10553). Anonymized data were obtained from the 2021 Sasakawa Sports Foundation National Sports-Life Survey of Children and Young People, which is a cross-sectional survey of a nationally representative sample of Japanese adolescents. We obtained only anonymous, data; therefore, informed consent was not required. The inclusion criterion was individuals aged 12-21 years living in Japan. There were no specific exclusion criteria. The participants were selected using a two-stage stratified random sampling method. Municipalities throughout Japan were stratified into 225 regions based on geographic areas and population sizes. The sample size for each region was allocated proportionally, according to the target age population. The participants from each region were selected using a Basic Resident Register and systematic random sampling. Data were collected using a self-reported questionnaire. Surveyors visited 3,000 participant homes and distributed paper-based questionnaires between June and July 2021. The participants were informed that the questionnaire was voluntary and anonymous. The surveyors revisited the homes and collected the questionnaires, after which consent was obtained by answering the questionnaire.

Health status

Self-rated health was measured using the question “How do you feel about your health?” The answers were “very healthy,” “healthy,” “not so healthy,” and “not healthy.” In the analysis, the four categories were dichotomized into healthy (very healthy/healthy) and unhealthy (not so healthy/not healthy) [2]. Depressive symptoms were assessed using the Japanese version of the Patient Health Questionnaire (PHQ)-8 [18]. It consists of eight questions, and the answer for each question ranges from “not at all" (zero points) to “nearly every day" (three points). The total scores for the eight items were calculated, with higher scores indicating more depressive symptoms. The participants were divided into two groups: non-depressive (≤nine points) and depressive (≥10 points) [18].

Sports participation

The participants indicated the type and frequency of up to five sports activities performed over the past year. Sports activities included extracurricular school and club activities but did not include activities in school classes or events, such as field days. The type of sports participation was described in an open-ended manner. The frequency of each sports activity was reported as numbers per year, month, or week, depending on the frequency. The number of activities per year was then summed to calculate the total number of sports participants. The sports participation levels were classified into four categories: high frequency (≥seven times/week (corresponding to ≥364 times/year)), moderate frequency (<seven , ≥three times/week (156-363 times/year)), low frequency (<three, >zero times/week (1-155 times/year)), and non-participation (zero times/week (0 times/year)). The classification in this study followed the literature and physical activity recommendations for adolescents [10,19].

Participant demographics

Sex, age, number of siblings, household income, breakfast consumption, screen time, and sleep duration were surveyed. The participants ages were divided into ≤17 years and ≥18 years. Answers to the number of siblings were dichotomized into 0 (single children) and ≥1 (those with siblings) [20]. The question on household income was answered by the participant’s parents. The answers were selected from 11 options (10 options from <two million yen/year to ≥10 million/year, and “I do not know”) and divided into <five million yen and ≥five million yen based on the mean annual income of the Japanese population [21]. Because 17% of the participants answered “I do not know,” we created a “do not know” category for the statistical analysis. Answers for breakfast frequency (almost every day, four to five days/week, two to three days/week, and rarely) were dichotomized into ≥six days/week (almost every day) and ≤five days/week [11]. Screen time was assessed using the question “How much time do you spend watching TV or DVDs, playing games (including TV, PC, and portable games), or using a smartphone outside of school or work on weekdays?” The answers were provided from eight options (seven options from <30 minutes/day to five hours/day, and “I do not know”) and dichotomized into <two hours and ≥two hours [10]. Hours of sleep were calculated using the participants’ self-reported wake-up times and bedtimes on weekdays and divided into ≥eight hours and <eight hours [10].

Statistical analysis

Categorical variables are expressed as numbers and percentages. Continuous variables are shown as median and quartile values owing to the non-normal distribution of the data. The association between self-rated health and sports participation was examined using univariate logistic regression analysis, wherein self-rated health was the objective variable and sports participation was the explanatory variable. The odds ratios (ORs) for varying participation levels were calculated using the non-participation group as a reference. Associations between self-rated health and other demographics were also assessed. Furthermore, multivariate logistic regression analysis was performed to examine independent association, with self-rated health as the objective variable and sports participation and all demographics as explanatory variables. Similarly, the associations among depressive symptoms, sports participation, and other participant demographics were assessed using univariate and multivariate logistic regression analyses. Statistical significance was set at P < 0.05.

## Results

The questionnaires were returned by 1663 adolescents (return rate: 55%). Of these, five were excluded because of incomplete answers regarding health status and sports participation. Data from the remaining 1,658 adolescents were used for analysis.

**Figure 1:**
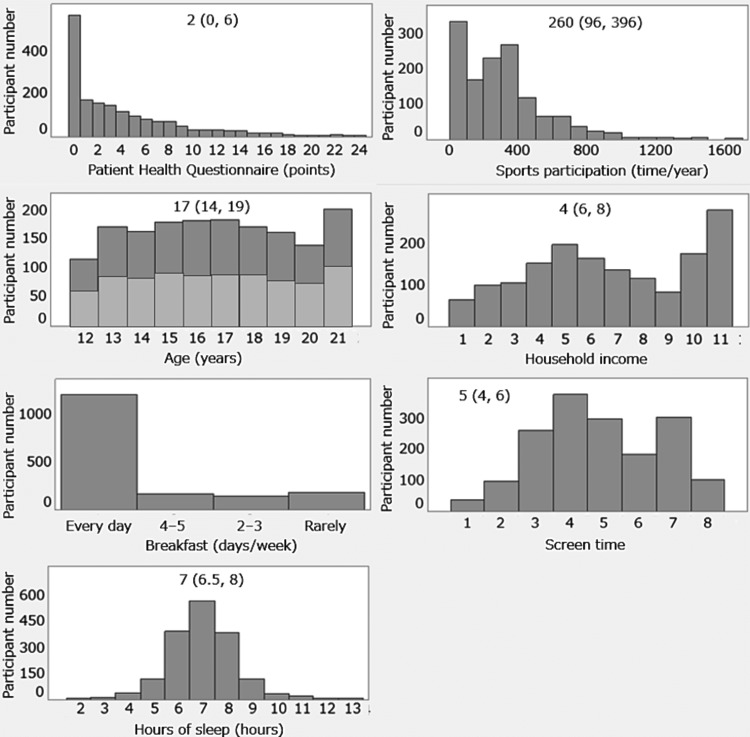
Detailed participant demographics (n = 1658). Numbers indicate median (25th, 75th percentiles) values. Answers of household income (million yen/year): 1, <2; 2, <3, ≥2; 3, <4, ≥3; 4, <5, ≥4; 5, <6, ≥5; 6, <7, ≥6; 7, <8, ≥7; 8, <9, ≥8; 9, <10, ≥9; 10, ≥10; 11, unknown. Answers of screen time (hours/day): 1, <0.5; 2, <1, ≥0.5; 3, <2, ≥1; 4, <3, ≥2; 5, <4, ≥3; 6, <5, ≥4; 7, ≥5; 8, unknown. Light and dark gray indicate male and female, respectively, in the "Age" figure.

Only 38 (2%) answered “not healthy,” while approximately one in five were classified in the unhealthy (not so healthy/not healthy) category (Table 1). The median PHQ-8 score was 2 (25th, 75th percentiles; 0, 6) points, with 174 (10%) classified as having depressive symptoms indicated by scores of ≥10 points (Table 1). While most participants engaged in sports activities at least once, 326 (19%) had not participated in any sports over the preceding year (Table 1). The participants were evenly distributed across sex and age groups, with a median age of 17 years (Table 1, Figure 1). The other demographic characteristics are shown in Table 1 and Figure 1.

**Table 1:** Participant demographics (n = 1658). PHQ, Patient Health Questionnaire. Number of participants with missing answers, n = 40^a^, 84^b^, 2^c^, 3^d^, 5^e^.

Item	n (%)
Self-rated health	
Healthy (very healthy/healthy)	1303 (79)
Very healthy	277 (17)
Healthy	1026 (62)
Unhealthy (not so healthy/not healthy)	355 (21)
Not so healthy	317 (19)
Not healthy	38 (2)
PHQ-8	
Non-depressive (≤9)	1484 (90)
Depressive (≥10)	174 (10)
Sports participation (times/week)	
High (≥7)	425 (26)
Moderate (<7, ≥3)	487 (29)
Low (<3, >0)	520 (25)
No (0)	326 (19)
Sex	
Male	837 (50)
Female	821 (50)
Age (years)	
≤17	988 (60)
≥18	670 (40)
Siblings^a^	
≥1	1340 (83)
0	278 (17)
Household income (yen/year)^b^	
≥5 million	871 (55)
<5 million	420 (27)
Unknown	283 (18)
Breakfast (days/week)^c^	
≥6	1208 (73)
≤5	448 (27)
Screen time (hours/day)^d^	
<2	390 (24)
≥2	1265 (76)
Hours of sleep (hours)^e^	
≥8	543 (33)
<8	1110 (67)

Among the four sports-participation groups, the proportion of unhealthy participants was the highest (n = 103, 32%) in the non-participation group (Table 2). The proportions decreased with an increasing frequency of sports activities. In the multivariate logistic regression analysis, the adjusted ORs were 0.45 (P < 0.001) in the high-frequency group and 0.46 (P < 0.001) in the moderate-frequency group, with the non-participation group as a reference. However, the difference between the non-participation and low-frequency groups was not significant (OR, 0.75; P = 0.10). In addition, the participants who ate breakfast ≥six days/week (OR, 0.43; P < 0.001) and who had screen times of <two hours/day (OR, 0.54; P < 0.001) were less likely to answer “unhealthy” (Table 2). Other demographics, such as sex, age, and hours of sleep, were not associated with self-rated health.

**Table 2:** Association of self-rated health with sports participation and participant demographics (n = 1658). OR: odds ratio; CI: confidence interval; Ref.: reference. ^a^n = 355. ^b^Model P < 0.001.

		Unhealthy^a^ n (%)	Univariate OR (95% CI)	P	Multivariate OR^b^ (95% CI)	P
Sports participation (times/week)	High	65 (15)	0.39 (0.27-0.56)	<0.001	0.45 (0.30-0.66)	<0.001
	Moderate	78 (16)	0.41 (0.29-0.58)	<0.001	0.46 (0.32-0.67)	<0.001
	Low	109 (26)	0.76 (0.55-1.04)	<0.10	0.75 (0.53-1.06)	0.10
	No	103 (32)	Ref.		Ref.	
Sex	Male	178 (21)	0.98 (0.78-1.24)	0.88	1.06 (0.82-1.37)	0.65
	Female	177 (22)	Ref.		Ref.	
Age (years)	≤17	185 (19)	0.67 (0.54-0.86)	0.001	1.20 (0.91-1.59)	0.20
	≥18	170 (25)	Ref.		Ref.	
Siblings	≥1	273 (20)	0.76 (0.56-1.03)	0.08	0.92 (0.66-1.27)	0.51
	0	70 (25)	Ref.		Ref.	
Household income (yen/year)	≥5 million	172 (20)	0.87 (0.65-1.15)	0.42	1.04 (0.77-1.40)	0.81
	Unknown	70 (25)	1.16 (0.81-1.65)	0.32	1.21 (0.83-1.75)	0.32
	<5 million	93 (22)	Ref.		Ref.	
Breakfast (days/week)	≥6	199 (16)	0.36 (0.29-0.47)	<0.001	0.43 (0.33-0.57)	<0.001
	≤5	156 (35)	Ref.		Ref.	
Screen time (hours/day)	<2	47 (12)	0.43 (0.31-0.60)	<0.001	0.54 (0.37-0.77)	<0.001
	≥2	307 (24)	Ref.		Ref.	
Hours of sleep (hours)	≥8	111 (20)	0.92 (0.71-1.18)	0.50	0.87 (0.67-1.15)	0.33
	<8	243 (22)	Ref.		Ref	

The proportion of participants with depressive symptoms was the highest (n = 45, 14%) in the non-participation group (Table 3). A U-shaped relationship was found, with the moderate-frequency group having the lowest prevalence of depression (n = 34, 7%). In the multivariate logistic regression analysis, only the moderate-frequency group showed a significant association, with an OR of 0.52 (P = 0.01). However, the differences between the high- and low-frequency groups and the non-participation group were not significant. Similar to the results of self-rated health, the participants who ate breakfast ≥six days/week (OR, 0.52; P < 0.001) and who had screen times of <two hours/day (OR, 0.42; P = 0.001) were less likely to report depressive symptoms (Table 2). Furthermore, the OR of the male sex was 0.55 (P < 0.001).

**Table 3:** Association of depressive symptoms with sports participation and participant demographics (n = 1658). OR: odds ratio; CI: confidence interval; Ref.: reference. ^a^n = 174. ^b^Model P < 0.001.

		Depressive^a^ n (%)	Univariate OR (95% CI)	P	Multivariate OR^b^ (95% CI)	P
Sports participation (times/week)	High	45 (11)	0.74 (0.48-1.15)	0.18	0.89 (0.56-1.41)	0.62
	Moderate	34 (7)	0.47 (0.29-0.75)	0.002	0.52 (0.31-0.87)	0.01
	Low	50 (12)	0.84 (0.55-1.30)	0.44	0.91 (0.56-1.48)	0.71
	No	45 (14)	Ref.		Ref.	
Sex	Male	66 (8)	0.57 (0.41-0.78)	<0.001	0.55 (0.39-0.78)	<0.001
	Female	108 (13)	Ref.		Ref.	
Age (years)	≤17	100 (10)	0.91 (0.66-1.25)	0.55	1.39 (0.95-2.02)	0.86
	≥18	74 (11)	Ref.		Ref.	
Siblings	≥1	132 (10)	0.76 (0.51-1.13)	0.17	0.81 (0.53-1.24)	0.33
	0	35 (13)	Ref.		Ref.	
Household income (yen/year)	≥5 million	87 (10)	1.05 (0.71-1.56)	0.79	1.30 (0.86-1.96)	0.21
	Unknown	37 (13)	1.42 (0.89-2.30)	0.14	1.63 (0.99-2.66)	0.05
	<5 million	40 (9)	Ref.		Ref.	
Breakfast (days/week)	≥6	101 (8)	0.48 (0.34-0.66)	<0.001	0.52 (0.36-0.74)	<0.001
	≤5	72 (16)	Ref.		Ref.	
Screen time (hours/day)	<2	20 (5)	0.39 (0.24-0.63)	<0.001	0.42 (0.25-0.71)	0.001
	≥2	154 (12)	Ref.		Ref.	
Hours of sleep (hours)	≥8	47 (9)	0.39 (0.24-0.63)	<0.001	0.80 (0.55-1.16)	0.24
	<8	127 (11)	Ref.		Ref.	

## Discussion

In a nationwide survey of Japanese adolescents, this study showed that moderate- to high-frequency sports participation was associated with lower odds of poor self-rated health. Moderate-frequency sports participation was also associated with lower odds of depressive symptoms, whereas high-frequency sports participation was not. Furthermore, the participants with shorter screen times and regular breakfasts were less likely to report poor health. The results of our study highlight that optimal sports activities may vary depending on the target health outcomes. In addition, a multidimensional approach can effectively reduce the risk of poor health.

In this study, the proportions of participants in the unhealthy category were lower than participants engaging in sports activities ≥three times/week than in those with no sports activities. However, the participation of <three times/week did not have a positive effect. Using general physical activity as the predictor, a recent meta-analysis showed that even a low frequency of physical activity had a small positive effect [3]. However, a lower threshold of the effect remains unclear [3]. Few studies have assessed the association of self-rated health and sports participation, and our results agree with studies of Swedish and Slovak adolescents, in which sports participation of ≥three to four times/week was associated with good self-rated health, while participation of lower frequency was not [19,22]. The results of the current study also correspond with the World Health Organization’s physical activity recommendation, in which vigorous-intensity activities should be incorporated at least three times a week [23]. Therefore, adolescents may be recommended to engage in sports at a moderate frequency or more.

We found a U-shaped relationship between sports participation and depression, in which the proportions of participants in the unhealthy category were lower in the moderate-frequency groups but not lower in the high-frequency groups. Our study was similar to one done on Canadian adolescents, which highlighted the differential effects of sports participation on self-rated health and depression [13]. According to a recent systematic review, the frequency of sports participation was negatively associated with depressive symptoms; nonetheless, the effect was weak [9]. A similar magnitude of the protective effect of sports participation, regardless of the frequency, has also been reported [24,25]. Furthermore, our study agrees with a survey of Swiss adolescents that also showed a U-shaped relationship, in which more than twice the recommended sport duration was associated with poor mental health [26]. These results may be particularly pertinent in Japan, where spending a large number of hours in extracurricular sports clubs has long been an issue among junior high school and high school students [27]. Indeed, the Japan Sports Agency recently developed guidelines on extracurricular sports activities that recommended at least two days off per week [27]. Therefore, optimizing sports participation frequency may lower the risk of depressive symptoms in adolescents. Extracurricular sports club activities should include appropriate days off in line with the guidelines.

This study showed that shorter screen time and regular breakfast consumption were associated with reduced odds of poor self-rated health and depressive symptoms. Screen time is linked to sedentary time, visual problems, mental health, and communication skills and is one of the major determinants of adolescent health [6]. Breakfast frequency is commonly used as an indicator of diet behavior in adolescents [11]. Breakfast skipping is a risk factor for poor physical and mental health in the future [11]. Furthermore, poor health conditions often result from multiple lifestyle factors. The results of our study are in line with those of previous studies and indicate that a multidimensional approach may be necessary to effectively prevent poor health in adolescents [6,28].

The strength of this study is that participants were recruited using a nationwide random sampling method. Therefore, the results likely reflect the health statuses of overall Japanese adolescents. Another strength is that we surveyed both self-rated health and depressive symptoms, enabling evaluation of the differential associations between sports participation and the health outcomes. However, this study also had several limitations. First, a causal association between sports participation and health status could not be determined because of the cross-sectional nature of this study. Nevertheless, other longitudinal studies have shown that participation in sports during early adolescence predicts lower depressive and anxiety symptoms in late adolescence and young adulthood because it offers an opportunity for physical activity and community involvement [9]. Second, although we surveyed a wide range of personal, lifestyle, and socioeconomic variables, several confounding factors, such as school satisfaction, were not included in our multivariate analysis [29]. Third, the questionnaire survey was conducted in 2021 during the coronavirus disease 2019 pandemic. Since physical activity and health statuses during the pandemic may have differed from those during non-pandemic periods, the results of our study may not apply to adolescents in the post-pandemic period [30]. Finally, the response rate of the survey was 55%. If individuals who were severely unhealthy or exhibited pronounced symptoms of depression were less likely to participate in the study, the prevalence of unhealthiness or depression might have been underestimated.

## Conclusions

Approximately 20% of the participants reported poor self-rated health. Furthermore, 10% had depressive symptoms. Moderate-to high-frequency sports activities are associated with a reduced risk of poor self-rated health among Japanese adolescents. However, only moderate-frequency activities were associated with a reduced risk of depression. Participation in optimal sports activities may effectively lower the risk of poor health in adolescents.
